# Supplementary material for: A Single Cell but Many Different Transcripts: A Journey into the World of Long Non-Coding RNAs
Source: Int J Mol Sci. 2020 Jan 1;21(1):302. doi: 10.3390/ijms21010302 (PMC6982300; doi:10.3390/ijms21010302)
Supplement: Supplementary file 1 [file ijms-21-00302-s001.zip › ijms-662665-suppl/Table S1.html]

**Table S1.** Small peptides coded by lncRNAs and their function.

| Species | Gene Symbol | Gene ID | Peptide length | Function | Description | Reference |
| --- | --- | --- | --- | --- | --- | --- |
| *Homo sapiens* | SPAAR | ENSG00000235387 | 90 | Muscle and cancer-related (oncogenic) | Negatively regulates mTORC1 activation and inhibits muscle regeneration | [1] |
|  | MYMX | ENSG00000262179 | 84 | Muscle-related | Regulates muscle development and muscle cell fusion | [2] |
|  | HOXB-AS3 | ENSG00000233101 | 53 | Cancer-related (tumor-suppressive) | Suppresses colon cancer aerobic glycolysis by inhibiting hnRNP A1-dependent PKM splicing | [3] |
|  | NBDY | ENSG00000204272 | 71 | Cancer-related and others | Involved in mRNA processing and negatively regulates P-body association | [4] |
| *Mus musculus* | Mrln | ENSMUSG00000019933 | 46 | Muscle-related | Interacts with SERCA (calcium-ATPase) and inhibits calcium reuptake into the sarcoplasmic reticulum | [5] |
|  | Strit1 | ENSMUSG00000103476 | 34 | Muscle-related | Enhances SERCA activity and calcium reuptake into the sarcoplasmic reticulum | [6] |
|  | Spaar | ENSMUSG00000028475 | 75 | Muscle and cancer-related (oncogenic) | Negatively regulates mTORC1 activation and inhibits muscle regeneration | [1] |
|  | Mymx | ENSMUSG00000079471 | 84 | Muscle-related | Regulates muscle development and muscle cell fusion | [2][7] |
| Danio Rerio | apela | ENSDARG00000094729 | 58 | Others | Activates G protein-coupled apelin receptor (APJ)/APJ signaling and promotes cell movement during gastrulation | [8] |
| *Drosophila melanogaster* | tal-1A/2A/3A/AA | FBgn0087003 | 11 and 32 | Others | Activates the transcription factor responsible for cuticle formation | [9] |
|  | SclB | FBgn0266492 | 28 and 29 | Muscle-related | Regulates calcium transport and muscle contraction | [10] |
|  | pgc | FBgn0016053 | 71 | Others | Represses CTD2 serine phosphorylation in germline progenitor cells | [11] |
| *Glycine max* | ENOD40A/B | SOY:0001919 | 12 and 24 | Others | Interacts with sucrose synthase and is required for plant–bacteria symbiotic interactions | [12] |
