# Supplementary material for: A Single Cell but Many Different Transcripts: A Journey into the World of Long Non-Coding RNAs
Source: Int J Mol Sci. 2020 Jan 1;21(1):302. doi: 10.3390/ijms21010302 (PMC6982300; doi:10.3390/ijms21010302)
Supplement: Supplementary file 1 [file ijms-21-00302-s001.zip › ijms-662665-suppl/Table S1.pdf]

Table S1. Small peptides coded by lncRNAs and their function.

Species	Gene Symbol	Gene ID	Peptide length	Function	Description	Reference
Homo sapiens	SPAAR	ENSG00000235387	90	Muscle and cancer-related (oncogenic)	Negatively regulates mTORC1 activation and inhibits muscle regeneration	[1]
	MYMX	ENSG00000262179	84	Muscle-related	Regulates muscle development and muscle cell fusion	[2]
	HOXB-AS3	ENSG00000233101	53	Cancer-related (tumor-suppressive)	Suppresses colon cancer aerobic glycolysis by inhibiting hnRNP A1-dependent PKM splicing	[3]
	NBDY	ENSG00000204272	71	Cancer-related and others	Involved in mRNA processing and negatively regulates P-body association	[4]
Mus musculus	Mrln	ENSMUSG00000019933	46	Muscle-related	Interacts with SERCA (calcium-ATPase) and inhibits calcium reuptake into the sarcoplasmic reticulum	[5]
	Strit1	ENSMUSG00000103476	34	Muscle-related	Enhances SERCA activity and calcium reuptake into the sarcoplasmic reticulum	[6]
	Spaar	ENSMUSG00000028475	75	Muscle and cancer-related (oncogenic)	Negatively regulates mTORC1 activation and inhibits muscle regeneration	[1]
	Mymx	ENSMUSG00000079471	84	Muscle-related	Regulates muscle development and muscle cell fusion	[2][7]
Danio Rerio	apela	ENSDARG00000094729	58	Others	Activates G protein-coupled apelin receptor (APJ)/APJ signaling and promotes cell movement during gastrulation	[8]
Drosophila melanogaster	tal-1A/2A/3A/AA	FBgn0087003	11 and 32	Others	Activates the transcription factor responsible for cuticle formation	[9]
	SclB	FBgn0266492	28 and 29	Muscle-related	Regulates calcium transport and muscle contraction	[10]
	pgc	FBgn0016053	71	Others	Represses CTD2 serine phosphorylation in germline progenitor cells	[11]
Glycine max	ENOD40A/B	SOY:0001919	12 and 24	Others	Interacts with sucrose synthase and is required for plant–bacteria symbiotic interactions	[12]
