# Supplementary material for: A Single Cell but Many Different Transcripts: A Journey into the World of Long Non-Coding RNAs
Source: Int J Mol Sci. 2020 Jan 1;21(1):302. doi: 10.3390/ijms21010302 (PMC6982300; doi:10.3390/ijms21010302)
Supplement: Supplementary file 1 [file ijms-21-00302-s001.zip › ijms-662665-suppl/Table S2.html]

|  |
| --- |
| **Table S2.** List of lncRNAs with a validated function.  This table contains a list of lncRNAs divided into groups depending on their known function. The references to the papers validating the lncRNA function are easily accessible by clicking the numbers near the name of the gene while clicking on the name itself will link to the ncbi page for that gene.  Data has been obtained from the database LncBook[0]. |

| Function | Validated lncRNAs |
| --- | --- |
| ceRNA | APF[1], ATB[2][3][4][5], BC032469[6], BGLT3[7], CARL[8], CASC2[9], CAT104[10], CBR3-AS1[11], CCAT1[12][13][14][15][16], CCDC144NL-AS1[17], CD99P1[18], CDKN2B-AS1[19], CDR1-AS[20], CERNA1[21], CERNA2[22], CERNA3[23], CHRF[24][25], CRNDE[26][27], CTB-89H12.4[28], CTD-3080P12.3[6], CYTOR[29][30], DLEU1[31], DUBR[32], FER1L4[33][34], FLJ90757[35], GAS5[36][37][38][39][40], H19[41][42][43][44][45][46][47][48][49][50][51][52][53], HNF1A-AS1[54], HOST2[22], HOTAIR[55][56][57][58][59][60][61][62][63][64][65], HOXA11-AS[66], HULC[67][68][69], KIAA1614-AS1[32], KRASP1[70], LA16c-313D11.11[71], LINC00052[72], LINC00115[73], LINC00161[74], LINC00240[75], LINC00319[76], LINC00882[32], LINC00941[77], LINC00974[78], LINC01234[10], LINC01262[79], LINC01772[80], LINC01826[81], linc-223[82], LINCMD1[83][84], LINC-ROR[85][86][87][88][89][90], lncARSR[91], LNCRI[92][93], lncRNA-ATB[2][3], lncRNA-DLEU1[94], lncRNA-FER1L4[95], lnc-SCA7[96], LOC283663[35], LOC338651[35], MAGI1-IT1[17], MALAT1[97][98][99][100][101][102][103][104][105][106][107][108], MEG3[109][110][111][73][112], MHENCR[113], MIAT[114][115][116][73], MINCR[117], MIR31HG[118], MVIH[119], NCK1-DT[120], NEAT1[121][122][123][124][125], PARAL1[126], PCA3[127], PCAT1[128], PCGEM1[129][130], POIR[131], PTCSC3[132], PTENP1[133][134][135], RMRP[136], RP11-457M11.2[137], RP11-838N2.4[138], RSU1P2[139], SLC26A4-AS1[17], SNHG14[140], SNHG6[141], STXBP5-AS1[10], TGFB2-OT1[142], TMSB4[143], TUG1[144][28][145][146], TUSC7[147][148], UCA1[149][150][151][152][153][154][155][63][156][157], ucoo2kmd.1[158], UFC1[159], Unigene56159[160], XIST[161][162][163][164], ZFAS1[165][166] |
| Protein Localization | 7SL[167], MALAT1[168], NEAT1\_2[168], POU6F2-AS2[169] |
| RNAi | CDKN2B-AS1[170], MEG3[171], SEC24B-AS1[172] |
| Splicing Regulation | 51A[173], ABALON[174], BCYRN1[175], MALAT1[176][177][178][179][180][134][181][182][183], MIAT[184][185], NPPA-AS1[186][178], TPM1-AS[187], uc002yug.2[188], ZEB2-AS1[189] |
| Transcriptional Regulation | 116HG[190], 7SK[191][192][193][194][195], 91H[196], AGAP2-AS1[197], AIRN[198], AK294004[199], APOA1-AS[200], APOC1P1-3[201], APTR[202], ASncmtRNAs[203], ATXN8OS[204], B4GALT1-AS1[205], BAALC-AS1[206], BACE1-AS[207], BALR-6[208], BANCR[209], BC041488[43], BCAR4[210][211][212], BDNF-AS[213][214][190], BLACAT1[215], C5T1lncRNA[216], CADM1-AS1[217], CAMTA1-DT[218], CAR Intergenic 10[192], CARD8-AS1[219], CARMN[220], CASC11[221], CCAT1-L[222], CCAT2[223], CCDC26[224], CCEPR[225], CDKN1A-AS1[226], CDKN2B-AS1[227][228][229][230][231][232][233][234][235][236][237][238][239][184][181][240][190][241][242], CFTR-AS1[243], Chaer[244], CISTR[245], COL1A2-AS1[246], CRNDE[247][248], CTBP1-AS[249][250][251][207], CYP4A22-AS1[252], CYTOR[253][254], DACOR1[255], DALIR[256], DANCR[257][258][259][260], DANT1[261], DANT2[261], DBCCR1-003[262], DBET[263], DGCR5[264], DHFR upstream transcripts[265], DHRS4-AS1[266], DILC[267], DISC1FP1[268], DISC2[269], DLEU1[270][271], DLEU2[270][271], DLX6-AS1[272], DQ786243[273], EBIC[274], ELFN1-AS1[275], EMX2OS[276], ERICD[277], Evf2[278], EVI1[279], EWSAT1[280], FALEC[252][281], FAS-AS1[282], FENDRR[283][284][285], FEZF1-AS1[286][286], FGF10-AS1[287], FIRRE[288], FLICR[289], FMR1-AS1[290], FOXCUT[291], FOXD3-AS1[292], FTX[293][294], GAS5[295], GATA3-AS1[296], GClnc1[297], GDNF-AS1[298], GHRLOS[299], GIHCG[300], GNAS-AS1[301], GNG12-AS1[302], GPC3-AS1[303], GPR1-AS[304], GSTT1-AS1[305], H19[306][307][308][309][310][311][312][313][314][315][181][316], HAR1A[317], HAR1B[317], HAS2-AS1[318], HIT[319], HOTAIR[320][321][322][323][324][325][326][327][328][329][330][331][332][333][334][38][335][336][337][338][339][340][341][342][343][344][345][346][347][178][348][135][349][350][351], HOTAIRM1[352], HOTTIP[353][354][355][356][207], HOXA-AS2[357], HOXC-AS1[358], HTTAS\_v1[190], HULC[359][360][361][362][363], IFNG-AS1[364][365], IGF2-AS[366], IPW[367], IRAIN[368][369], JADRR[370], JPX[371], KCNQ1OT1[372][373][374], LALR[375], LINC00312[376], LINC00473[377], LINC00570[252], LINC00628[378], LINC00672[379], LINC00673[380], LINC00853[252], LINC00887[381], LINC01081[382], LINC01116[383], LINC01133[384], LINC01158[385], LINC01191[386], LINC01207[387], LINC01562[388], LINC01629[381], LINC02574[389], LINC02575[389], LINC-PINT[390], lincRNA-LALR1[391], lincRNA-p21[134], LINC-ROR[392][393], Lnc34a[394], lnc-DILC[267], lnc-NKX2-3-1[395], LNCPRESS2[396], lncRNA-CD244[305], LncRNA-LALR1[391], lnc-RTN4R-1[395], LNCSRLR[397], LOC100887755[252], LOC389023[214], LRP1-AS[398], LRRC3DN[399], LUADT1[400], LUARIS[401], LUCAT1[402], LUNAR1[403], MAFTRR[404], MALAT1[405][406][407][408][409][410][411][412][413][414][415], MAPT-AS1[416], MEG3[417][418][419][420][421][422], MHRT[423], MIF-AS1[424], MINCR[425][426], MIR2052HG[427], MIR210HG[381], MRUL[428], MYCNOS[429][430], NALT1[431], NBAT1[432][433], NBR2[434][435][435], ncNRFR[436], ncR-PAR[437], NCRUPAR[437], NEAT1[438][439][440], NFIA-AS1[441][442], NKX2-2-AS1[443], NORAD[444], NRAV[445], NRIR[446], PACERR[447], PAN[448][449], PANDAR[450][451][180], PARTICL[452][453], PAUPAR[454][455], PCA3[456][457], PCAT1[458][459][251], PCAT6[252], PCGEM1[460][461], PCNA-AS1[462][463][464], PEG13[465], PICSAR[466], PINCR[467], PINK1-AS[468], PISRT1[469], PLUT[470], POU3F3[385], Ppp1r1b[471], PR antisense transcripts[472], PRINS[473], PRNCR1[474][460], PTCSC2[475], PTENP1[476], PTENP1-AS[476], PTPRJ-AS1[477], PVT1[478][479][480][481][482][483][484], RAD51-AS1[485], RASSF1-AS1[486], RBM5-AS1[487], RMST[488], RNA-a[190], ROCR[489], RP11-359E19.2[43], RRP1B[490], SBF2-AS1[491], SCAANT1[492], SCHLAP1[493], snaR[494], SNHG20[495], SOCS2-AS1[496], SOX2-OT[497][498], SOX9-AS1[489], SPRY4-IT1[499][500], SRA[501][502][348], TARID[503][504], TEX41[505], TH2LCRR[506], THRIL[507], TMPOP2[274], TP53COR1[508][509], TRAF3IP2-AS1[510], TRERNA1[252], TSIX[511], TUG1[512][513][514][515][516], TUNAR[517], TUSC7[518], uc.338[519], uc.345[520], UCA1[521][522][523][524][525][526], UFC1[527], UNMIBC[528], WFDC21P[529], WSPAR[530], WT1-AS[531][532], XIST[533][534][535][536][537], YAM1[538][538], ZFAS1[539][540] |
| Translational Control | BACE1-AS[541][178][542], BCYRN1[543], BTG3-AS1[544], FILNC1[545], HIF1A-AS2[546], LNCPRESS1[547], MALAT1[548], MSNP1AS[549], NORAD[550], PPP1R12A-AS1[551], PVT1[552][553], RBM15-AS1[554], SATB2-AS1[555], SNHG6[556], UCHL1-AS1[168] |
