# Supplementary material for: A Single Cell but Many Different Transcripts: A Journey into the World of Long Non-Coding RNAs
Source: Int J Mol Sci. 2020 Jan 1;21(1):302. doi: 10.3390/ijms21010302 (PMC6982300; doi:10.3390/ijms21010302)
Supplement: Supplementary file 1 [file ijms-21-00302-s001.zip › ijms-662665-suppl/Table S3.pdf]

Table S3. Interactions between lncRNAs and other molecules.

This table contains a list of human lncRNAs divided into groups depending on their known interactions with DNA, Proteins or other RNA molecules. The references to the papers validating the lncRNA interactions are easily accessible by clicking the numbers near the name of the gene while clicking on the name itself will link to the ncbi page for that gene.

Data has been obtained from the database EVLncRNAs[0].

	Binding	Co-Expression	Regulation
**RNA-DNA**	AL121845.1[1], CDKN2B-AS1[2], CYTOR[3], HOTAIR[4][5], KCNQ1OT1[6][7], Khps1[8], MALAT1[9], NEAT1[10], PARTICL[11], RBM5-AS1[12], RNY1[13], UCA1[14], ULK4P2[15], WT1-AS[16]	FRLnc1[17], HOTAIRM1[18], MEG3[19]	AATBC[20], AC021078.1[21], alpha-280/250[22], BACE1-AS[23], BANCR[24][25], BOK-AS1[26], BRCA1[27], C1orf74[28], CAR Intergenic 10[29], CCDC26[30], CCEPR[31], CDKN2B-AS1[32], DANCR[37], DHFR upstream transcripts[38], DHRS4-AS1[39], EGFR-AS1[40], ENST00000414355[41], FALEC[42], FOXC2-AS1[43], FOXCUT[44], GAS5[45], H19[46], HIF1A-AS1[49], HOTAIR[50], HOTAIRM1[61], HOTTIP[62], HOXA11-AS[66], HOXA-AS[67], HULC[68], IRAIN[69], KCNQ1OT1[70], LINC00261[71], LINC00312[72], LINC00951[73], LINC00964[74], LINC01024[75], LINC01207[76], LINC-ROR[77], Lnc34a[78], lncARSR[79], lncRNA-422[57], lncRNA-AK058803[80], lncRNA-ATB[81], LOC401317[82], LUADT1[83], MALAT1[84], MDC1-AS1[89], MEG3[90], MINCR[94], ncRNACCND1[95], NCRUPAR[96], NRAV[97], OR3A4P[98], PANDAR[99], PCA3[100], PCAT1[101][102], PICSAR[103], PTCSC3[104], PVT1[105], SNCG[108], SNED1[109], SNHG12[110], SPRY4-IT1[111], SRA1[112], TRAF3IP2-AS1[113],

			TUG1[21], TUNAR[114], TUSC7[115], ZEB1-AS1[116], ZFAS1[117]
**RNA-Protein**	Alu lncRNAs[118], AOC4P[119], ASncmtRNAs[120], BCYRN1[121], BCYRN1P2[128], CDKN2B-AS1[32], CTBP1-AS[134], CYTOR[135], DHFR upstream transcripts[38], EWSAT1[136], FENDRR[137], GAS5[138], GSTT1-AS1[141], H19[142], HEIH[146], HOTAIR[50], HOTTIP[62], HOXA-AS2[153], KCNQ1OT1[154], KIR antisense lncRNA[155], LINC01207[76], lincDR1[137], lincGARS[137], lincMLKN1[137], LINC-ROR[156], lincSFPQ[137], lnc13[157], LUADT1[83], MALAT1[158], MEG3[90], MIAT[162], MT1JP[163], ncRNACCND1[112], NEAT1[164], NKILA[171], NRON[172], OIP5-AS1[173], PAN[174], PARTICL[11], PCAT1[175], PCNA-AS1[176], pncRNA-D[177], Prion-associated RNAs[178], PVT1[179][107], RN7SK[180], RN7SL1[204], RRP1B[207], SARCC[208], SPRY4-IT1[209], SRA1[210], TINCR[216], TUG1[137], UCA1[14], WFDC21P[219], WSPAR[220][221], XIST[150]	AC100861.1[222], BCYRN1[125], CCAT1[223], CDKN2B-AS1[224], CPS1-IT1[225], DLEU1[226], H19[227], HOTAIR[228], HOTTIP[229], MVIH[230], UCA1[231]	AB073614[232], AF339813[233], AFAP1-AS1[234][235], AOC4P[236], APOA1-AS[237], ASncmtRNAs[120], AT102202[238], BACE1-AS[239], BALR-6[240], BANCR[241][242], BRCA1[27], BX647187 [243], CBR3-AS1[244], CCAL[245], CDKN2B-AS1[246][247], DACOR1[248], DANCR[249], DBET[250], DBH-AS1[251], DLEU2[252], DLX6-AS1[253], FALEC[254], FAM30A[255], FENDRR[256], FTX[257], GAS1RR[258], GAS5[167], H19[265], HBB[274], HIF2PUT[275], HNF1A-AS1[276], HOTAIR[50], HOTTIP[285][286], HOXA-AS2[153], HULC[287], IFNG-AS1[291][292], IL7R[293], ILF3[81], LINC00635[294], LINC00668[295], LINC00982[296], LINC01158[297], LINC01426[298], linc-ITGB1[299], LINC-ROR[300], LINK-A[301], lnc-bc060912[302], lnc-DILC[303], LUNAR1[304], MALAT1[305], MEG3[167], MIAT[321], MIR31HG[322][323], MT1DP[324], MT1JP[163], NALT1[325], NBAT1[326], NBR2[327], NCRUPAR[328], NEAT1[329][330], NORAD[331], NRON[332][167], PACERR[333], PCAT5[334], PCOTH[335], POU6F2-AS2[336], PRAL[337], PRINS[338], PVT1[339][340], RAD51-AS1[341], RGMB-AS1[342], RMST[343], RN7SK[344], SAMMSON[352], SFTA3[353], SIRT1-AS[354], SNHG15[355], SRA1[356][215], TP53COR1[357][358], TP73-AS1[359], TSIX[360], TUG1[218][361], TUSC7[362][363], UCA1[364],

			UCHL1-AS1[371], VIM2P[372], WSPAR[221], XIST[373], ZFAS1[374]
**RNA-RNA**	BACE1-AS[375], BDNF-AS[376][167], CASC2[377], CCAT1[378][379], CD99P1[380], CTD-3080P12.3[381], GAS5[382][264], H19[383], HNF1A-AS1[278], HULC[38], LINC01613[380], lncARSR[79], LOC100129973[386], MALAT1[387], MVIH[388], NUTF2P3[389], PCGEM1[390], PINK1-AS[391], RN7SL1[167], SIRT1-AS[392], TGFB2-OT1[393][394], TUSC7[363], UCA1[395], ZFAS1[117]	ATXN8OS[397], BACE1-AS[375], C5T1lncRNA[398], CADM1[399], CDKN2B-AS1[132], CDR1-AS[400], CYP4A22-AS1[401], DLEU1[226], EMX2OS[402], FALEC[403], FTX[257], H19[404], HOTAIR[407], HOTAIRM1[132], HOTTIP[132][408], HOXA11-AS[409], LINC00570[401], LINC00853[401], LINC00974[410], LINC01612[411], LSINCT5[412], MEG3[413], ncRNA-a6[401], NUTF2P3[389], PCAT1[175], PCAT6[401], PRINS[414], PTENP1[415], RBM4[416], RBM5-AS1[12], SRA1[417][213], XLOC_010235[411], ZEB2-AS1[418]	asOct4-pg5[419], B4GALT1-AS1[420], BACE1-AS[239][375], BANCR[241], BCYRN1[127], CADM1[421], CYP4A22-AS1[403], EGFLAM-AS1[422], EMX2OS[423], FAS-AS1[424], FER1L4[425], FOXCUT[426], FTX[427], GAS5[428], H19[268], HIF2PUT[434], HOTAIR[435], HOTTIP[63], HULC[289][290], ICR[442], LINC00570[403], LINC-ROR[300][443], lncRNA-ATB[444], LUNAR1[445], MALAT1[435], MEG3[452], MIAT[321], MT-ND5[456], MT-ND6[456], ncRNA-a6[403], NEAT1[457], OIP5-AS1[173], PCAT6[403], PCGEM1[458], RN7SK[459], SKP2[460], SOX2-OT[461], SPRY4-IT1[286], TP53COR1[358], TRERNA1[403][462], TSIX[360], TUG1[463], UCA1[464], UFC1[465], WRAP53[466][467], XIST[468]
